# To Our Subscribers

**Published:** 1891-07

**Authors:** 


### Editorial.

### TO OUR SUBSCRIBERS.

After considerable hesitation the proprietors of The Journal have decided to change its title page to the present form.

We hope you will consider that it *improves* rather than *mars* the appearance of The Journal, and that you will not think that an old, familiar form has been detroyed—at a loss.

